# Impact of Polymer Membrane Properties on the Removal of Pharmaceuticals

**DOI:** 10.3390/membranes12020150

**Published:** 2022-01-26

**Authors:** Renata Żyłła, Magdalena Foszpańczyk, Irena Kamińska, Marcin Kudzin, Jacek Balcerzak, Stanisław Ledakowicz

**Affiliations:** 1 Łukasiewicz Research Network-Textile Research Institute, ul. Brzezińska 5/15, 92-103 Łódź, Poland; magdalena.foszpanczyk@iw.lukasiewicz.gov.pl (M.F.); irena.kaminska@iw.lukasiewicz.gov.pl (I.K.); marcin.kudzin@iw.lukasiewicz.gov.pl (M.K.); 2 Department of Molecular Engineering, Faculty of Process and Environmental Engineering, Lodz University of Technology, Wólczańska 213, 93-005 Łódź, Poland; jacek.balcerzak@p.lodz.pl; 3 Department of Bioprocess Engineering, Faculty of Process and Environmental Engineering, Lodz University of Technology, ul. Wólczańska 213, 93-005 Łódź, Poland; stanislaw.ledakowicz@p.lodz.pl

**Keywords:** nanofiltration, pharmaceuticals, hazardous materials, membrane filtration, amoxicillin, diclofenac, ibuprofen, tetracycline, salicylic acid, acetylsalicylic acid

## Abstract

The influence of various factors on the removal efficiency of selected pharmaceuticals by membrane filtration was investigated. Several commercial polymer membranes were used for nanofiltration (NF) from various manufacturers. The studies were conducted for ibuprofen (IBF), amoxicillin (AMX), diclofenac (DCF), tetracycline (TRC), salicylic acid (SA) and acetylsalicylic acid (ASA). The influence of the structure and properties of the tested compounds on the retention coefficient and filtration rate was investigated. The influence of pH on the filtration parameters was also checked. The properties of selected membranes influencing the retention of pharmaceuticals and filtrate flux were analysed. An extensive analysis of the retention coefficients dependence on the contact angle and surface free energy was performed. It was found that there is a correlation between the hydrophilicity of the membrane and the effectiveness and efficiency of the membrane. As the contact angle of membrane increased, the flow rate of the filtrate stream increased, while the retention coefficient decreased. The studies showed that the best separation efficiency was achieved for compounds with a molecular weight (MW) greater than 300 g/mol. During the filtration of pharmaceuticals with MW ranging from 300 to 450 g/mol, the type of membrane used practically did not affect the filtration efficiency and a high degree of retention was achieved. In the case of low MW molecules (SA and ASA), a significant decrease in the separation efficiency during the process was noted.

## 1. Introduction

The subject of wastewater treatment and water recovery is constantly facing new challenges. The development of analytical methods enabled the identification of new threats resulting from the presence of trace amounts of hazardous substances in the natural environment due to human activity. Such substances include pharmaceuticals and hormones. Concerns about the presence of pharmaceutically active compounds in drinking water and surface waters have been increasing since the late 1990s [1,2,3,4] The constantly increasing demand for pharmaceuticals and their consumption, combined with incomplete metabolism in the human body, have led to an increase in concentration in wastewater and surface waters to which they are discharged. Research conducted around the world shows a fairly common presence of pharmaceuticals in surface waters as well as in wastewater and sludge [3,4,5,6,7,8].Their concentration in tested water samples ranges from ng/dm^3^ to μg/dm^3^ and it is predicted that with the growing human population and the greater dependence of modern societies on pharmaceuticals, their concentration in water reservoirs will increase [9,10,11]. Conventional wastewater treatment plants are not fully effective in removing pharmaceutical contaminants [12,13,14]. Disposal rates for pharmaceuticals in conventional wastewater treatment plants are generally around 40–60% [15]. Secondary sewage discharged from wastewater treatment plants becomes the main source of pharmaceutical micropollutants [12]. It has been shown that water treatment plants are not fully effective in removing anthropogenic organic micropollutants because their main function is to remove natural organic matter and microorganisms [16]. Although the presence of these compounds in the environment corresponds to low concentration levels, their continuous flow from wastewater treatment plants or direct discharge into natural river beds may pose a long-term potential threat to aquatic and terrestrial ecosystems [15]. Therefore, there is a need to implement efficient technologies in wastewater treatment plants that ensure the elimination of biodegradable pharmaceuticals from wastewater before their introduction into the aquatic environment [15].

A technique that ensures effective removal of chemical compounds from a solution is membrane filtration [5,17,18,19,20]. Disposal of pharmaceuticals using advanced membrane technology has therefore become of particular interest in water/wastewater treatment and water reuse [10,19,21,22].

The mechanism of pharmaceutical retention on the membrane is complex and depends on many factors: membrane properties (surface hydrophobicity, surface charge, pore size), physicochemical properties of the solute (molecular weight, charge, hydrophobicity), the chemical composition of the matrix in which the compound is dissolved, as well as filtration parameters (pressure, permeation rate, cross flow velocity) [23,24].

Nanofiltration (NF) and reverse osmosis (RO) offer a very strong possibility of removal for most organic micropollutants, since the molecular weight of these pollutants is often around 200–300 g/mol, and the molecular weight cut-off (MWCO) values of NF membranes are also often in this range (for RO membranes, the MWCO values are even lower). However, removal of some organic micropollutants is still incomplete and traces may still be detected in the permeate of NF and RO installations [25].

From previous reports in the literature on the removal of pharmaceuticals from water by NF, it can be concluded that the electrostatic interactions of a charged molecule with the membrane, and the retention of the molecule on the membrane due to its size, are of key importance in the separation process (this applies to molecules whose molar mass is greater than the MWCO value). In contrast, in the case of hydrophobic uncharged molecules, the adsorption of molecules on the membrane plays a major role [10,24,26].

It is widely recognized that the membrane charge is an important factor influencing the separation efficiency of charged molecules [27]. It is strongly correlated with the transport of molecules through the boundary layer and in the membrane itself. Consequently, it becomes necessary to understand the charge change of the NF membrane to achieve the highest efficiency in the process. NF membranes absorb electric charge through several mechanisms. When they come into contact with the electrolyte solution, the functional groups of the membrane dissociate depending on the pH of the solution. Other mechanisms include adsorption of ions from solution, adsorption of polyelectrolytes, ionic surfactants and charged macromolecules [28,29].

The retention value strongly depends on the pH of the feed water, since both the surface charge of the membrane and the organic solute change with pH (via dissociation of functional groups as a function of pKa) [25,30]. In the case of NF polyamide membranes, the presence of carboxyl and amine functional groups on their active layer make the NF membranes susceptible to ionization with changes in pH. Ionization of such groups is reflected in the variability of the zeta potential of the surface membrane with increasing and decreasing pH [28,31,32,33,34]. Numerous studies have shown that nanofiltration membranes are negatively charged at pH >4 as a result of deprotonation of acid functional groups (-COO−) of the polyamide top layer [11,26,27,35,36]. Membranes can have different zeta potentials depending on the degree of cross-linking of the polyamide and the number of functional groups on the surface. Hence, an increase in pH causes a significant increase in the separation efficiency of the membrane [32].

Another property of the membrane that has a significant impact on the separation mechanism of organic compounds is its hydrophilicity/hydrophobicity. The parameter that determines the degree of hydrophobicity of a given surface is its contact angle, which depends on the chemical structure of the membrane [37,38].

An important phenomenon affecting the separation mechanism is the adsorption of particles on the membrane due to hydrophobic interactions [11,39]. During the first steps of nanofiltration, some compounds are strongly adsorbed on the membrane. However, it has been shown that as filtration progresses, there is a decrease in retention of these compounds, possibly caused by saturation of membrane active sites and diffusion of compounds through the membrane after equilibrium has been reached [23,39]. Adsorption is the result of various interactions between the solute and the membrane. These include electrostatic, hydrophobic and some specific interactions (e.g., hydrogen bonds) [11,18,40]. It has been shown that more hydrophobic and less soluble compounds have a greater tendency to adsorb on the membrane surface [39,41].

The separation mechanism of pharmaceuticals that dominates in the process depends on the type of membrane, the chemical composition of the water matrix and the type of removing compound [25,28,31,32,33,34,39].

Knowledge of the mechanisms taking place during the membrane filtration process helps to avoid the problems related to the reduction of retention and filtrate flux during the process. Expanding knowledge on the effective disposal of pharmaceuticals will increase the safety of aquatic ecosystems. This study is an analysis of the influence of the membrane type and the pharmaceutical structure on the efficiency and effectiveness of nanofiltration for several commercial membranes. The article complements the research in previously published scientific papers [5,16,41,42,43,44,45]. The influence of the contact angle of the membranes on the filtration efficiency was analyzed from a new perspective. Although many researchers have focused on the mechanisms of solute transport in NF membranes including electrostatic interaction, hydrophobic interaction and size exclusion, still further studies are required to understand the mechanism that is affected by solute properties, membrane parameters, feed water composition and operating parameters [19].

## 2. Methodology

### 2.1. Materials

Analytical grade salicylic acid (SA) and acetylsalicylic acid (ASA), diclofenac (DCF), ibuprofen (IBU), amoxicillin (AMX) and tetracycline (hydrochloride) (TRC) were purchased from Sigma–Aldrich (St. Louis, MO, USA). For the tests, solutions with a molar concentration of the active substance equal to 5 × 10^−4^ mol/dm^3^ were used. Table 1 shows the structural formulas of the above-mentioned compounds and their basic properties.

The solutions of the tested compounds were acidic or slightly acidic. For comparison purposes, some tests were performed at pH = 8. NaOH solution was used to correct the pH.

### 2.2. Research Equipment

The nanofiltration process was carried out with the crossflow method at a constant flow rate of liquid inside the system of 2 dm^3^/min at 30 °C [46]. The tests were performed at a pressure of 1.0 MPa. The starting volume of the sewage was 3 dm^3^, the solution was concentrated to a volume of 1.5 dm^3^ (1:2).

Seven flat sheet membranes with an area of approximately 314 cm^2^ each were selected for the tests, the parameters of which are summarized in Table 2. Literature data on zeta potential values at pH = 8 and the isoelectric point for the selected membranes are summarized in a Table 3. A new, unused membrane was used for each experiment. Before use, each membrane was subjected to a pressure of 1.5 MPa for 50 min at ambient temperature (from 25 to 29 °C) during the filtration of pure deionized water. After 50 min, the pressure was reduced to 1.0 MPa and the flow rate of the filtrate stream was measured for 10 min at 30 °C.

### 2.3. Analytical Methods

#### 2.3.1. FTIR—Fourier Transform Infrared Spectroscopy

The IR spectra were made on a Jasco FTIR spectrometer equipped with a Pike ATR adapter with thermostatic control in the range from 400–4000 cm^−1^. 

#### 2.3.2. LC—Liquid Chromatography

All chemical compounds were monitored by determining the concentration using Shimadzu Nexera-i LC-2040C 3D plus apparatus equipped with Kinetex C18 column (2.6 μm). The 0.1% formic acid water solution (A) and the 0.1% formic acid acetonitrile solution (B) were used as eluents. The columns were thermo-stated at 40 °C. The injection volume was 10 μL. In the case of SA, IBF and TRC analysis, the flow rate was 0.4 mL min^−1^ and for the remaining compounds the flow rate was set at 0.5 mL min^−1^. Detection of compounds was carried out at 263, 270, 275, 300 and 360 nm for IBF, AMX, DCF, SA and TRC, respectively.

In the case of tests carried out at pH = 8, concentration measurements on LC were performed with use of standard curves prepared on the basis of standard pharmaceutical aqueous solutions at pH = 8.

#### 2.3.3. SEM Microscopic Analysis

The SEM microscopic examination was performed on a TESCAN VEGA3 scanning electron microscope (Tescan, Czech Republic). A magnification of 20,000× *g* was used to study the surface topography of membranes.

#### 2.3.4. Determination of Contact Angle and Free Surface Energy (FSE)

The contact angle was studied by the goniometric method using a PGX Goniometer (FIBRO Systems, Sweden). The tests were carried out at 22.4 ± 2 °C and relative air humidity 35 ± 2%. Contact angles for each test were determined using three standard liquids:

Diiodomethane, DIM (γL = 50.80 mJ/m^2^: γLLW = 50.80 mJ/m^2^, γL+ = 0 mJ/m^2^, γL− = 0 mJ/m^2^).

Water, W (γL = 72. 80 mJ/m^2^: γLLW = 21.80 mJ/m^2^, γL+ = 25.50 mJ/m^2^, γL− = 25.50 mJ/m^2^).

Formamide, F (γL = 58.00 mJ/m^2^: γLLW = 39.00 mJ/m^2,^ γL+ = 2.28 mJ/m^2^, γL− = 39.60 mJ/m^2^).

In determining the mean value of the contact angle, 10 repetitions of the measurements were made. Based on the determined contact angles, free surface energy γS of the membrane was calculated using the acid–base method, identifying the proportion of dispersion component γSLW and acid–base component γSAB in total free surface energy (γS = γSLW + γSAB).

#### 2.3.5. X-Ray Photoelectron Spectroscopy (XPS)

The analyses of the surface composition of membranes were carried out with the use of the AXIS Ultra photoelectron spectrometer by Kratos Analytical Ltd. The source of photoelectron emission from the sample surface (down to approx. 5 nm deep) was X radiation generated by the Al anode with monochromator (Kα line with an energy of 1486.6 eV). All XPS spectra were made using a charge neutralizer due to the insulating nature of the sample material. The analysis was performed on 3 to 6 fields with an area of 700 × 300 μm each, on the surface of each sample. The parameters of the XPS analysis are presented in Table 4.

## 3. Results and Discussion

### 3.1. The Influence of Molecule Structure on Their Retention

**Figure 1:**
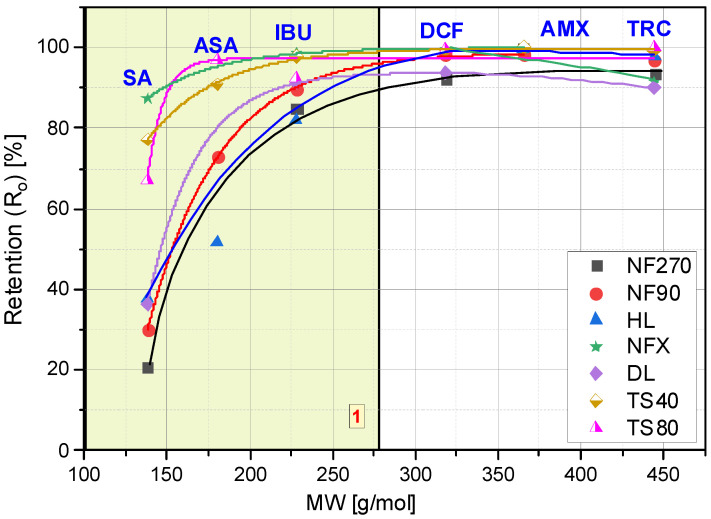
Dependence of the initial retention coefficient (R_o_) on the molar mass (MW) of the tested compounds.

**Figure 2:**
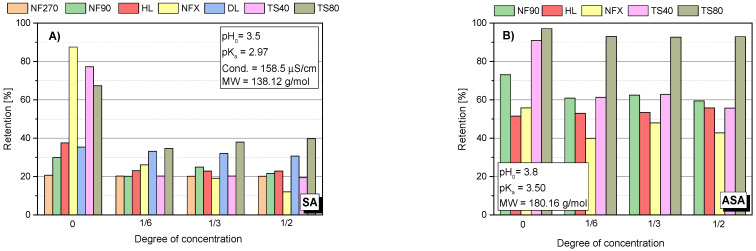

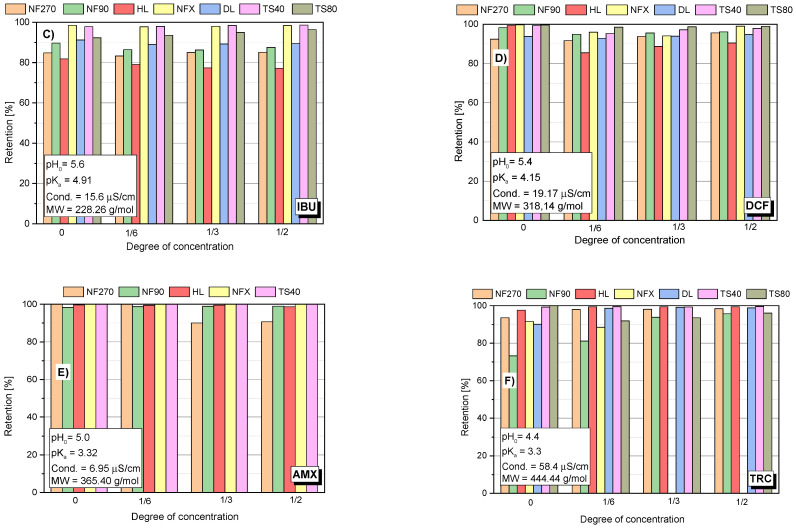
Dependency of the retention of the tested compound: (**A**) SA; (**B**) ASA; (**C**) IBU; (**D**) DCF; (**E**) AMX; (**F**) TRC; on the concentration degree of the aqueous solution for the tested membranes; pressure 1 MPa; temp. 30 °C.

Several factors affect the pharmaceutical retention simultaneously during the nanofiltration process. In addition to the structure, charge and size of the retained compound, the type of membrane plays an important role: its charge, hydrophilicity, roughness and MWCO value. The compound structure, as well as the type of membrane determines the separation mechanism on the membrane. The identification of this mechanism requires the observation of the changes in parameters of the filtration process over time. Figure 1 shows the initial values of the retention coefficients (R_o_) of the selected compounds for various membranes. Figure 2A–F show the retention coefficients (R) of the selected compounds (SA, ASA, IBU, DCF, AMX and TRC) depending on the filtrate concentration degree during the process. The measurements were taken at the beginning of the process (zerodegree concentration), firstly, obtaining 500 mL of filtrate from 3000 mL of the initial bath (concentration degree 1/6), and secondly, obtaining 1000 mL of filtrate (concentration degree 1/3) and 1500 mL of filtrate (concentration degree 1/2). The tests were performed with the use of several commercial polymer membranes (NF270; NF90; HL; DL; NFX; TS40 and TS80). The experiments were performed at a solution pH range from 3.5 to 5.6, depending on the type of compound.

The lowest values for the retention coefficient were for SA, the molecule that had the lowest molecular weight among the tested compounds (Figure 1 and Figure 2A). Its molecular weight is 138 g/mol and is lower than the MWCO value of the polymer membranes used (from 150 to 400 Da). ASA is similar in structure to SA, it has a carboxyl group attached to the molecule and its molecular weight is 180 g/mol. The higher molecular weight of ASA and the presence of a spatial obstruction improved the separation efficiency on the membrane (Figure 1 and Figure 2B).

Similar studies on the dependence of retention on molar mass were carried out by [25]. The research was carried out for low molecular weight organic acids of 50 to 125 g/mol. It was shown that with the increase in the molar mass of these compounds, the efficiency of their separation on the Trisep TS80 and Desal HL membranes increased [25]. Veriefde and co-workers [25] showed that their retention is determined not only by the effect of charges, but also by steric interactions. The retention of benzoic acid was lower than that of malonic and lactic acid, despite its higher molecular weight. The authors argued that it was probably due to the lower three-dimensional configuration of the phenyl group of benzoic acid compared to the linear carbon backbones of lactic and malonic acids [25]. However, some researchers have emphasized that although molecular weight (MW) is the most frequently used parameter to reflect particle size, in the case of charged and hydrophobic compounds it may prove to be a weak indicator [16].

IBU and DCF particles have a molecular weight ranging from 200 to 300 g/mol. Both compounds showed significantly higher retention coefficients when compared to SA and ASA, although for membranes with higher MWCO (NF270, NF90) they did not exceed 95%. The literature also reports high IBU and DCF retention rates [5,16,41,42].

The best separation efficiency was obtained for compounds with MW greater than 300 g/mol (DCF, AMX and TRC) (Figure 2D,F). For the MW range from 138 g/mol (SA) to 318 g/mol (DCF) (see yellow area (1)) there is a clear dependence of the retention rate on the molecule size. In this area, the influence of the type of membrane on the separation efficiency can be clearly seen. Similar conclusions were reached by [44]. The authors studied the retention of various hormones and pharmaceuticals (including tetracycline) using the nanofiltration membrane NF200 (Film Tech Corp., Minneapolis, MN, USA).

Among the tested compounds, the highest retention rates were obtained for AMX. Only an insignificant drop was observed for the loose NF270 membrane. Shahtalebi and co-workers [55], achieved similarly high efficiency for a spiral NF membrane (Film Tec NF40). Benitez and co-workers [56], achieved about 98% AMX retention in the filtrate for the HL membrane at a pressure of 2.0 MPa. Since the amount of MWCO of the NF membrane is lower than the amoxicillin molecular weight, the amoxicillin molecules are almost rejected while the organic molecules having molecular weight lower than MWCO of NF membrane can penetrate the membrane [55,57]. The experiments with the NF270 membrane obtained lower AMX retention values (approx. 80%) than for DCF and IBU (studies were carried out for pure compounds). The molecular weight cut-off of the NF270 membrane was about 300 g/mol. However, the rejection rates of some drugs whose molecular weight was larger than 300 g/mol were still below 90% [57].

The analysis of the changes in the retention coefficient values of the selected compounds during the filtration process made it possible to obtain information on the separation mechanism (Figure 2A–F). In the case of low molecular weight molecules (SA and ASA), a significant decrease in the separation efficiency during the process was observed (Figure 1 and Figure 2A–B). An initial significant drop in retention was followed by an equilibrium step in which the retention (R) value was constant throughout the filtration period. For low molecular weight molecules (SA and ASA), the separation efficiency generally depended on the type of membrane used, particularly at the start of the process. For most tested membranes, the SA retention coefficient at equilibrium was about 20%. The exception was the TS80 and DL membranes. The equilibrium retention value for the TS80 membrane was approx. 40% and for the DL membrane approx. 30%. Such a drop in retention is characteristic of the adsorption phenomenon. The pH of the solutions of the tested acids ranged from 3.5 to 3.8. This is the pH range at which many polyamide membranes have an isoelectric point. The surface of most membranes is either uncharged or slightly negatively charged. Kim and co-workers [43], noted slight reductions in SA retention during the nanofiltration process using a polyamide membrane NE4040-70 (SAEHAN Corp., Seoul, Korea), made of trimesoyl chloride and m-phenylene diamine (MWCO = 200 Da; zeta potential above −30 eV; contact angle 43°). However, the authors used a less concentrated SA solution (1 mg/dm3) and a smaller volume of the starting solution (500 mL). Accumulated permeate volume was 60 mL. The research was conducted at pH 7 (with a phosphate buffer). The higher pH allowed for better separation due to electrostatic repulsion between the SA and the membrane [43].

In the case of ASA a decrease in the retention rate was noted for the NF90 and TS 40 membranes (Figure 2B). For NFX and HL membranes the initial retention rate was the lowest compared to other membranes and was in the range of 50–60%. These values did not change during the process.

In the case of IBU, DCF and AMX molecules, the retention rates did not change significantly during the process. IBU and DCF are negatively charged in the aqueous environment and most authors justify the separation of these compounds by electrostatic repulsion from the negatively charged membrane surface [10,16,58,59]. There are works that document significant adsorption of IBU on a membrane, including a hydrophilic membrane [58]. In its neutral form, IBU is highly hydrophobic, which is reflected in its high log Kow value, and this observed adsorption can probably be attributed to the hydrophobic interactions between IBU and hydrophobic domains in the polymer matrix of the membrane [58]. It should be noted that in this study the research was carried out for the sodium salt of IBU and DCF, which significantly improves the solubility of the compounds.

In the case of high molecular weight TRC (approx. 450 g/mol), there was a slight increase in the retention coefficient (R) during the concentration of the solution, which is probably due to the fact that TRC is deposited on the membrane surface and creates an additional spatial barrier for such large molecules.

Research on the use of nanofiltration for TRC removal was carried out for various nanofiltration membranes, both commercial ones: NF90 (Dow FilmTech, Bean Station, TN, USA) [60]; TFC-SR2 and NF-SR3 (Koch, Wichita, KS, USA) [45]; NF200 (Film Tech Corp., Minneapolis, MN, USA) [44], as well as made by the authors [61]. The results of the research described in this publication supplement the research conducted by other authors.

### 3.2. The Influence of Molecule Structure on Filtrate Flux

**Figure 3:**
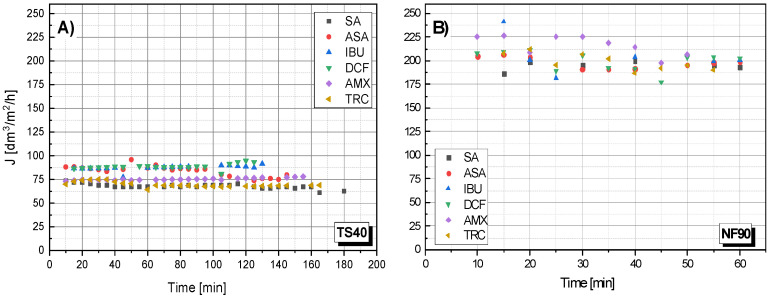
The dependence of the filtrate stream flow rate (J) on the filtration time of various compounds: (**A**) for the TS40 membrane; (**B**) for NF90 membrane; pressure 1.0 MPa, temperature 30 °C.

Figure 3 shows the dependency filtrate flow rate on the filtration time for the TS40 and NF90 membranes. In general, there was no clear effect of molecule size on the filtration rate. All the processes were similar. However, it seems that large molecules (TRC) filter slightly slower. Unfortunately, the obtained results do not allow for drawing clear conclusions in this regard.

The NF90 membrane was characterized by a higher filtrate flux than the data described in the literature [50], for the NF90 membrane, obtained pure water permeability at 46.9 L/h m^2^.

### 3.3. Influence of pH on Retention Coefficient

**Figure 4:**
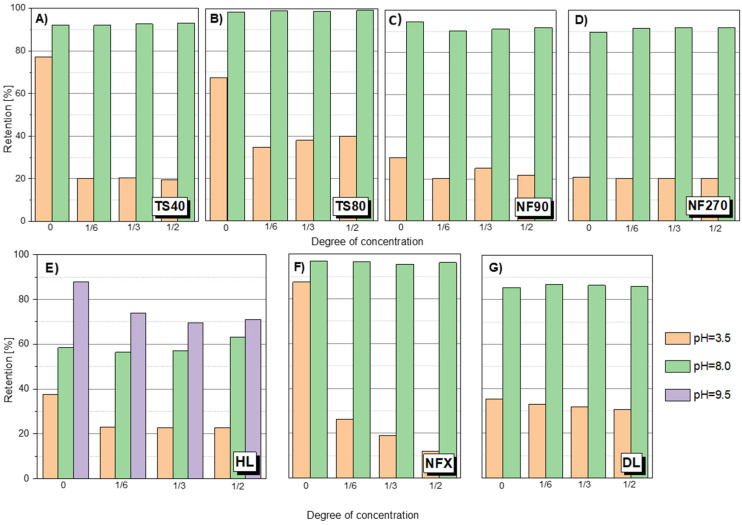
SA retention at different pH for different membranes: (**A**) TS40 membrane; (**B**) TS80 membrane; (**C**) NF90 membrane; (**D**) NF270 membrane; (**E**) HL membrane; (**F**) NFX membrane; (**G**) DL membrane; pressure 1.0 MPa, temperature 30 °C.

**Figure 5:**
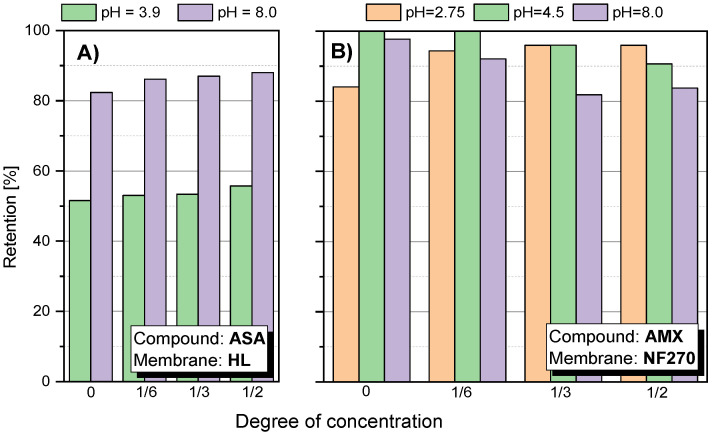
Retention factor depending on the concentration of the concentrate: (**A**) for ASA using HL membrane; (**B**) for AMX using NF270 membrane; pressure 1.0 MPa; temperature 30 °C.

There was a pronounced pH effect on the retention coefficient value of the tested compounds. Tests for different pH values were performed for SA, ASA and AMX. Figure 4 shows the dependence of SA retention on the solution concentration at different pH values and for different membranes. It was found that at a slightly alkaline pH (pH = 8.0) the separation efficiency was much higher than at pH = 3.5 in all tested cases. The pKa of SA is 2.97. Among the tested compounds, it has the highest conductivity value (158 μS/cm) and has two dissociating groups. In an alkaline environment, SA is practically dissociated in the form of anions. Similar results were obtained for ASA (Figure 5A). As the pH increased, the retention of ASA increased.

Changes in membrane surface charge is a key factor in predicting membrane behavior and is strongly dependent on the pH of the feed solution. The interaction of functional groups on the polymer surface structure is pH dependent. In the case of NF polyamide membranes, the presence of carboxyl and amine functional groups on their active layer makes NF membranes susceptible to ionization with changes in pH. The ionization of such groups is reflected in the variability of the zeta potential of the membrane surface with increasing and decreasing pH [31,33,34]. The negative zeta potential of polymer membranes results in more negatively charged membranes and favors stronger electrostatic interactions between dissociated functional groups from the material surface [12]. At pH range 3–4, the zeta potential for most of them is close to zero [11,36,51,52,53,54]. At pH 8, the zeta potential value increases significantly. Temperature was not found to have a significant influence on the zeta potential of the membrane in the temperature range of 20–34 °C [27].

The carboxyl groups (-COO−) that may be present on polyamide membranes are slightly acidic and do not dissociate at low pH [32]. At an alkaline pH the membranes are negatively charged due to the dissociation of carbonyl groups of the polyamide top layer. Membranes can have different zeta potential depending on the degree of cross-linking of the polyamide and the number of functional groups on the surface. Hence, an increase in pH causes a significant increase in the separation efficiency on the membrane.

The effect of pH on organic acid retention was investigated by [25]. The retention of all acids (weighing from 50 to 125 g/mol) in Milli-Q water at pH 8 was greater than 93%. Acid retention at pH 8 also increases with the increasing molecular weight of the acids tested.

For the HL membrane a slight increase in retention is noticeable at 50% concentration of the stock solution at pH 8.0. A similar phenomenon can be observed for ASA (Figure 5A). However, it may be due to the influence of factors other than just the membrane charge increase. It has been reported that such electrostatic interactions can constrict the pores of the membrane as the pH increases [12,25,28]. Figure 4E shows the SA retention results for the HL membrane at pH 9.5. There was a tendency to decrease retention during solution concentration, which may indicate partial destruction and loosening of the membrane structure as a result of the pH being too high.

A slight increase in retention during the concentration process at pH 8 was noticeable for the HL membrane. Based on the data collected in the literature, it can be concluded that the HL membrane, like the NF270, has a bimodal pore diameter distribution. Two pore sizes prevail in the structure of the membrane, which is confirmed by the presence of two clearly separated peaks on the pore size distribution curve [62,63,64,65]. Pore sizes were determined in this study by the modified examination method based on the transport of specific substances (markers). Summarizing data from several studies, it can be determined that the HL membrane has the most pores with a size from 0.71 to 1.03 nm in diameter, and relatively fewer large pores with a size from 1.3 to 2.0 nm. Perhaps the changes in the pore diameter distribution improves SA and ASA retention when concentrating the solution. Although the NF270 membrane has also been reported to be loose with a bimodal pore diameter distribution [63], the increase in retention was not as pronounced. Perhaps this is due to the fact that the HL membrane is clearly less electronegative than the NF270 membrane. Therefore, at pH 8 the pore narrowing of the HL membrane has a noticeable effect. It is probable that for the NF270 membrane the effect of electrostatic repulsion is completely dominant. The analysis of the effect of the pore size of the membranes is quite difficult because some membranes have a bimodal pore diameter distribution (HL, NF270, TS80), others do not (NF90) [62,65]. For example, the NF80 membrane has a bimodal pore diameter distribution, but has a much smaller pore sizes, which means that the membrane generally provides high retention of the compounds [38,65]. Additionally, the TS80 and TS40 membranes are one of the most electronegative (Table 3).

Due to the complexity of the phenomenon, more research is needed in this area, as many factors influence the mechanism of the separation process. It should be noted that for the HL membrane an increase in flux was observed with increasing pH, and for the NF270 membrane, the pH had no effect on the flux (Table 3).

Figure 5B shows the value of the AMX retention factor for the NF270 membrane at three pH values: 2.75; 4.5 and 8.0. In the case of low pH 2.75, the initial retention was relatively lower than the values obtained at pH 4.5 and 8.0. Nevertheless, the value of R increased during the process. At pH 8.0, the opposite effect was observed. In the first phase of the process, R was about 98–100%, while the process was running, the R value decreased to about 80%. This is likely due to a change in the filtration mechanism that is influenced by several factors. AMX is a relatively strong organic acid (pKa = 3.23). However, the electrolytic conductivity of AMX is the lowest among the compounds tested (approx. 7 μS/cm). Taking into account the MW of AMX, the spatial blocking mechanism dominates, despite the fact that the NF270 membrane is one of the most electronegative membranes among the studied.

### 3.4. Influence of pH on Filtrate Flux

The dependence of the filtrate flux on pH was investigated for the SA solution as a standard substance. There was an increase in filtrate flux for the HL, TS40 and TS80 membranes (Table 5). In the case of DL and NFX membranes, the expressed effect of pH on the flux was not observed. In the case of NF270 and NF90 a slight increase in flow was observed.

### 3.5. Influence of Membrane Properties on Filtration Parameters

The type of membrane was important for the separation of compounds with MW below 300 g/mol (Figure 1 and Figure 2). The effect of membrane properties on filtration parameters was investigated. Among other things, the influence of membrane hydrophilicity on the separation efficiency of selected compounds was analyzed. The values of the contact angle for the tested polymer membranes are summarized in Table 6.

**Figure 6:**
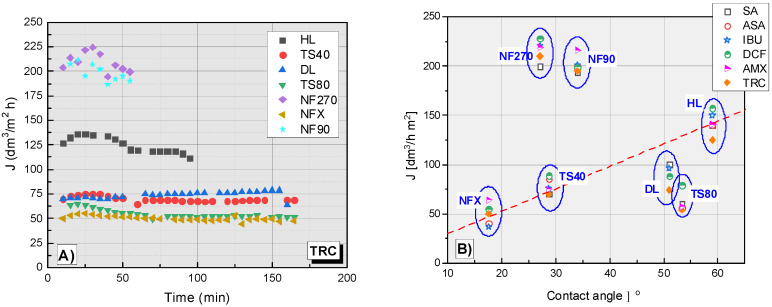
(**A**) The dependence of the filtrate flux on the filtration time of the TRC solution. (**B**) The dependence of the filtrate flux on the contact angle of tested membrane for the tested compounds; contact angle for DL (ϴ = 51°) [37].

Figure 6A shows the dependence of the filtrate flux on the filtration time of the TRC solution. In the TRC filtration process, the highest flux filtrate was obtained for the NF270 and NF90 membranes (Figure 6A). The filtrate flux values for these membranes ranged from approx. 200 to 225 dm^3^/m^2^ h. The HL membrane was characterized by an average filtration efficiency (approx. 125 dm^3^/m^2^ h) between the membranes with the highest efficiency (NF270, NF90) and the other tested membranes (TS40, TS80, NFX and DL), the filtrate flux of which was between 40-80 dm^3^/m^2^ h. The slowest filtration was on NFX and TS80 membranes. The DL and TS40 membranes showed similar performance, slightly better than the NFX and TS80.

Figure 6B shows a collective plot of the dependence of filtrate flux on the contact angle of different membranes during the filtration process. The contact angle of the membrane is one of the most important factors influencing the filtrate stream. A correlation has been observed between filtrate flux (J) and contact angle in the case of the NFX, TS40 and HL membranes. As the contact angle increases, the filtration rate increases. The TS80 membrane is slightly below the dependency line. In contrast, the NF90 and NF270 membranes are well above the curve. For the DL membrane, no contact angle measurements were made.

**Figure 7:**
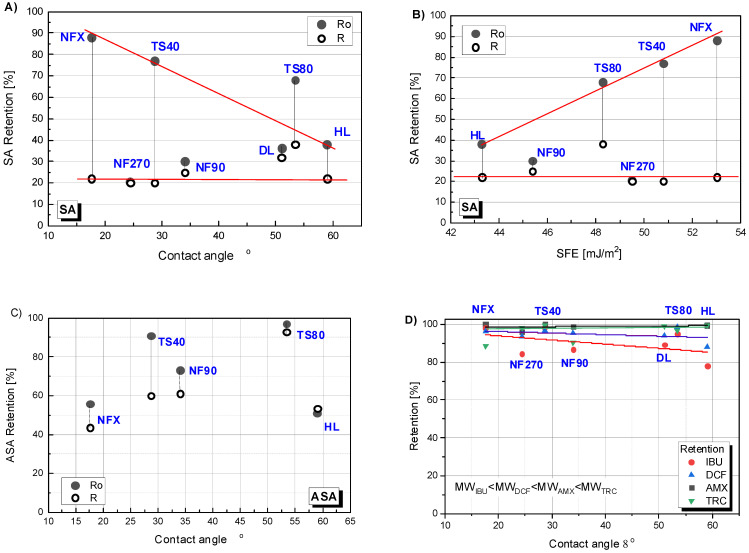
Retention dependence of selected compounds on the contact angle of polymer membranes: (**A**) for salicylic acid (SA); (**C**) for acetylsalicylic acid (ASA); (**D**) for ibuprofen (IBU), diclofenac (DCF), amoxicillin (AMX) and tetracycline (TRC); (**B**) Retention dependence of SA on surface free energy (SFE). R_o_—initial retention; R—average retention during the process; contact angle for DL (ϴ = 51°) [37].

The retention dependence on the contact angle is presented in Figure 7A,C,D. The figure shows two values of the retention coefficient: at the beginning of the process (Ro) (the value was significantly higher than the values obtained during the process) and the average value (R) for the concentration degree from 1/6 to 1/2 (in this range, R were very close to each other except for the results obtained for TS80). For easier interpretation of the results for all cases, a linear theoretical correlation curve was drawn. In Figure 7A it can be seen that the linear correlation between the initial SA (Ro) retention value and the contact angle for the NFX, TS40 and HL membranes fits well. The TS80 membrane clearly showed a higher initial retention than it would appear from the determined curve. An inverse linear correlation was noticed for initial SA retention and SEP of the HL, TS80, TS40 and NFX membranes (Figure 7B). The NF270 and NF90 membranes did not fit into any predetermined correlation. The results for ASA were difficult to interpret (Figure 7C). On Figure 7D the data for four compounds simultaneously: DCF, IBU, AMX and TRC, have been shown. Despite some scatter in the experimental results for IBU and TRC, a trend can be observed that the lower the molar mass of the test compound, the stronger the influence of the contact angle on the initial retention (Ro). However, the effect was less significant compared to SA. In the case of AMX and TRC molecules, there was no effect of contact angle on membrane retention. Probably, because the main influence on the separation of AMX and TRC was spatial blockage.

**Figure 8:**
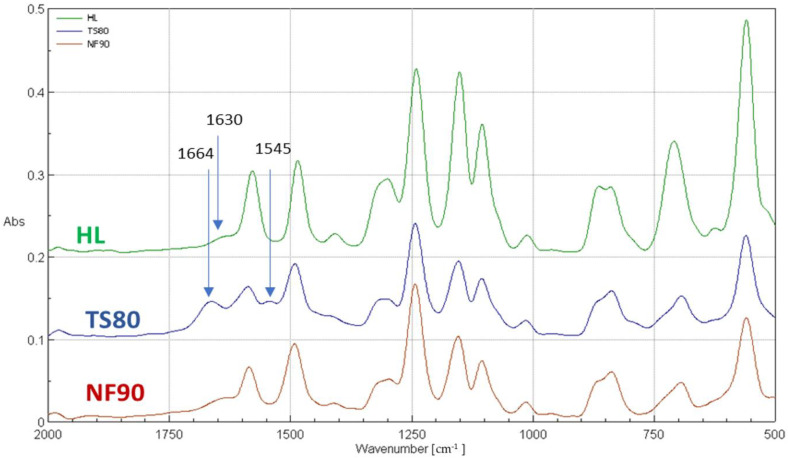
FTIR spectrum for selected membranes for wavenumbers from 500 to 2000 cm^−1^.

**Figure 9:**
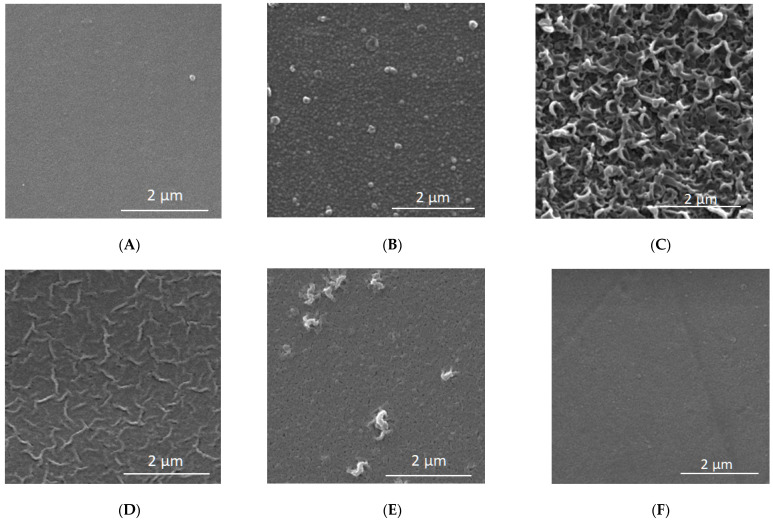
SEM microscopic images for selected membranes at 20,000 times magnification. (**A**) Clean HL; (**B**) Clean TS40; (**C**) Clean TS80; (**D**) Clean NF90; (**E**) Clean NFX; (**F**) Clean NF270.

In order to correctly interpret the results, further studies were necessary using other measurement techniques such as FTIR (Figure 8, Table 7) and XPS (Table 8, Table 9). Both FTIR and XPS spectroscopic measurements can provide important chemical and elemental information. The literature data on the zeta potential (Table 2) and SEM microscopic photos (Figure 9) were also taken into account.

FTIR and XPS analyses were performed for the selected membranes: TS80, NF90 and HL. Figure 8 shows the FTIR spectra of selected membranes for the wave number ranging from 500 to 2000 cm^−1^. The FTIR spectrum includes bands of both the polyamide top layer as well as the polysulfone sublayer. Distinct peaks at 1586 cm^−1^ and 1485 cm^−1^ belonged to stretching vibrations in the plane of the aromatic ring [66]. The peak at the wavenumber of 1294 cm−1 was caused by the asymmetric stretching vibrations of the -SO_2_ groups. A strong peak at about 1152 cm^−1^ represented symmetrical vibrations stretching the -SO_2_ groups. The peak at about 1242 cm^−1^ was associated with asymmetric C-O-C bond vibration of the aryl-O-aryl group. These are characteristic peaks for polysulfone functional groups, confirming that all tested NF membranes have a polysulfone support layer [66].

The peaks at 1664 cm^-1^ and 1545 cm^-1^, which are unique to the polyamide skin top layer, were observed on the TS80 membrane spectrum [67] (Table 7). The 1664 cm^−1^ peak, which is usually identified as an amide I mode in a secondary amide group, may consist of contributions from the C=O stretching (largest contribution), the C–N stretching and the C–C–N deformation vibration [26,37,67]. The amide II band (1545 cm^−1^) is due to a motion combining both N–H (largest contribution) in-plane bending and the N–C stretching vibrations of the group –CO–NH– in its trans form [26,37,67]. The peaks at 1665 cm^−1^ and 1545 cm^−1^ were invisible for HL and NF90 membranes. Instead, a peak at 1630 cm^−1^ typical for poli(piperazineamide), was observed.

Based on the ratio of the surface areas of individual high-resolution elemental bands (narrow scan), taking into account individual X-ray sensitivity factors, a quantitative analysis (XPS) was performed for individual samples showing the relative share of elements, expressed in atomic %. In Table 8, average values of the atomic fraction of oxygen, nitrogen and carbon determined on the surface of the tested membranes are presented. XPS results showed that all the membrane surfaces contained predominantly oxygen, nitrogen and carbon. The analysis showed that the NF90 membrane had relatively less nitrogen and more oxygen than the other tested membranes (HL and TS80). The O/N ratio for the NF90 membrane was 1.66, while for the HL and TS80 membrane it was 1.42 and 1.51, respectively. The O/N ratio indicates the degree of cross-linking of polymer chains [69]. For an unmodified polyamide (PA) layer, the O/N ratio = 1 is attributed to the fully cross-linked PA layer, where each oxygen atom is bonded to a nitrogen atom in an amide bond. The O/N ratio = 2 is attributed to the linear PA layer with additional oxygen atoms in the free carboxylic groups [47]. Table 9 summarizes the components in the C 1s band for the analyzed membranes. The component responsible for carbon–carbon bonds (C-C/C-H, C=C) dominated. Comparative wide scans and C 1s region of an XPS spectrum of the membranes are included in the Supplementary Material.

**Figure 10:**
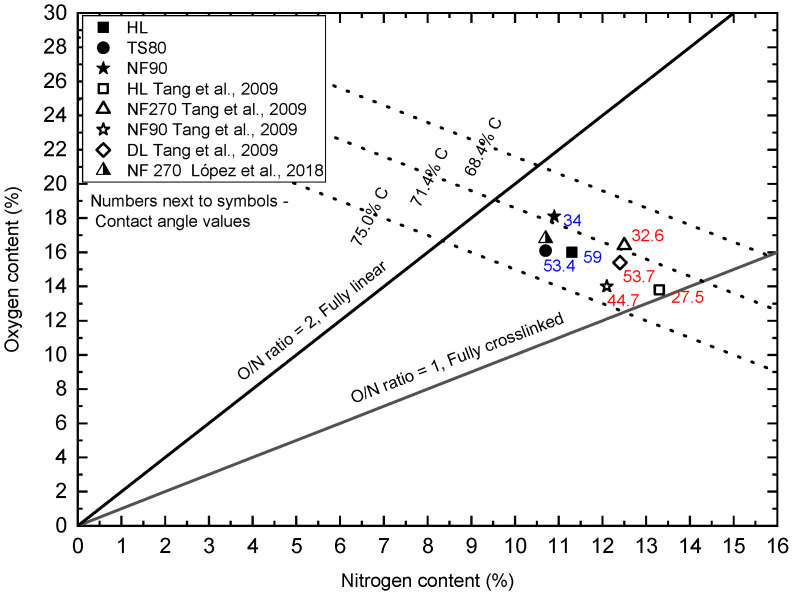
Oxygen versus nitrogen content from XPS survey scans for tested membranes: HL, TS80 and NF90. The figure includes the available literature data [47,68]. The figure shows the available values of the contact angle—the numbers next to the symbols.

Oxygen versus nitrogen content from XPS survey scans obtained for tested membranes: HL, TS80 and NF90 were plotted on the chart proposed by [37]. Figure 10 includes the available literature data [37,68]. The figure contains the theoretical carbon content lines (75.0, 71.4, and 68.4%) for fully aromatic polyamide (based on trimesoyl chloride and 1,3-benzenediamine) and poly(piperazinamide). For the fully cross-linked polymer the values are: 75.0% and 71.4% for fully aromatic polyamide and poly(piperazinamide), respectively. For the fully linear polymer the values are: 71.4% and 68.4% for fully aromatic polyamide and poly(piperazinamide), respectively [37,68].

The HL and NF270 membranes are characterized as smooth, loose and with a bimodal pore size distribution [35,62,63,64,65,70,71]. They are characterized as made of a uncoated semi-aromatic polyamide obtained by interfacial polymerization of trimesoyl chloride (TMC) and piperazine [37,47,53]. In the literature, the HL and NF270 membranes are considered to be some of the more hydrophilic [47,72]. In most studies, the contact angle (determined by the deposited drop method using clean water as a medium) for these membranes ranges from approx. 27 to approx. 36° [47,49,72,73]. Park [71] found the value of the contact angle for the HL membrane to be 15°. In our research, the HL membrane turned out to be the least hydrophilic (59°) among the tested membranes. The HL membrane had the typical white color for uncoated poly(piperzanamide) membranes or PVA coated ones, the same as NF270 membrane. The O/N ratio was clearly higher than that of Tang and co-workers [47], for the same carbon content (Figure 10). This may indicate a weaker cross-linking of the membrane, or some modification to the poly (piperazinamide) structure by the manufacturer. The modification may be supported by a higher contact angle and, at the same time, a lower zeta potential (the lowest SA retention coefficient at pH 8 among all membranes tested) [47]. The HL membrane is from the same manufacturer as the DL membrane (GE Osmonics) which according to Tang [37,47] has been characterized as modified poly(piperazinamide). There are works that show that the HL membrane is much more hydrophobic. Al-Amoudi and co-workers [74] determined that their HL membrane had a contact angle of 56.7°. Park and co-workers [71] in their earlier work determined the contact angle for the DESAL HL membrane to be 50.9°.

The NF90 membrane in most publications is characterized as a dense membrane made of fully aromatic polyamide [50,53,70]. Among the membranes tested, it usually shows a relatively low filtrate flux and a high retention rate [50]. The SEM screens shows that its surface is uneven and wavy [75]. The contact angle for NF90 membrane was 34o. In the literature, the contact angles for this membrane were usually higher, indicating lower hydrophilicity from 43 to 65.19° [50,51,52,73,76]. Our research shows that we received NF90 membranes from the manufacturer with characteristics similar to the NF270 membrane. Unfortunately, the processes and precise chemistry of producing commercial polymer membranes are proprietary, which greatly limits the understanding of membrane users about the physical and chemical properties of these membranes [53]. Moreover, the surfaces of some membranes are often modified by further treatment steps such as the application of a neutral hydrophilic coating layer to improve membrane performance in terms of permeability, rejection or contamination [37,47,53].

In the literature, membrane TS80 is classified as a dense, tight membrane with a high retention factor [25,77]. At the same time, comparing the contact angle values for selected membranes, the TS80 membrane turned out to be one of the least hydrophilic, relatively. Its contact angle was 53°, the value was similar to the literature data (48°), [42,78]. Based on the SEM microscopic images presented in Figure 9, it can be concluded that the TS80 membrane had the most developed surface topography compared to the other membranes. Our tests confirmed that the TS80 membrane is made of fully aromated polyamide (Figure 8).

The points for the TS40 membrane were placed on a line between the NFX and HL. The determined 28° contact angle was close to the value obtained by (34.5°) [35]. However, considering the value of the contact angle determined by Schmidt et al. (2020) the area of the TS40 points would coincide with the area of the DL membrane. The DL membrane is characterized as a poly(piperazinamide) modified additional coating [37]. It is considered more compact than the NF270 membranes [35].

A very unusual case is the NFX membrane which, apart from the worst retention rates, also showed the worst process efficiency. Typically, as the efficiency of the process increases, its efficiency decreases. In our analysis, it turned out to be the most hydrophilic membrane among the respondents. The contact angle of its surface was only 17.7° (Table 6). There are few publications devoted to this membrane [79,80,81].

Taking into account all the results, it can be assumed that the retention of pharmaceuticals was influenced by both the structure of the molecule present in the filtered medium and the type of membrane used. In general, this effect was noticeable for compounds with a mass below the MWCO of the membrane used. Compounds with molecular weights above 300 g/mol had high retention rates regardless of the filtration conditions. On the other hand, flux was mainly influenced by the type of membrane used, assuming that pure dilute aqueous solutions were filtered. It has been observed that probably in the case of acidic dilute aqueous solutions of pharmaceuticals with low molecular weight (below MWCO), the adsorption phenomenon is important in the initial stage of the filtration process. This process is likely to be dependent on the membrane hydrophilicity. Adsorption did not allow the compounds to be permanently retained on the hydrophilic membranes used in the experiments. After the process stabilized, most of the tested pharmaceuticals were transferred into the filtrate.

## 4. Conclusions

Seven polymer membranes with different surface structures were examined in the study. The research was carried out for six pharmaceuticals of different molar mass. The study analyzes the influence of the structure of the molecule of the removed compound on the efficiency and effectiveness of membrane filtration. In the MW range from 138 g/mol (SA) to 318 g/mol (DCF) there is a clear dependence of the retention factor on the molecule size of a given compound. For compounds with MW higher than 300 g/mol (AMX and TRC) the separation efficiency was close to 100%. The main separation mechanism for large molecules was probably trapping them in the spatial structure of the membrane. For smaller molecules, adsorption in the polymer structure of the membrane was important, which would appear from the clear difference between the retention value in the initial filtration process and the value during the concentration process. Most of the solutions had a low pH, close to the isoelectric point of the examined membranes. Therefore, the electrostatic interactions between the tested compounds and the membrane were weak. With the increase of pH, the value of the retention coefficients of the tested compounds increased. This was due to the fact that the surface charge of the membrane increased with the degree of dissociation of the negatively charged functional groups of the membrane.

From a filtrate flux point of view, the particle size of the pharmaceuticals removed was not of great importance, although a slight downward trend in flux was observed for high molecular weight compounds.

The influence of the membrane properties on the filtration efficiency was also analyzed. The obtained test results showed that the type of membrane has a significant impact on the filtrate flux as well as on the retention of removed compounds. The NFX membrane showed the worst performance in terms of flux. The best performance was obtained with the NF270 and NF90 membranes. The NF90 membrane showed many features of membranes made on the basis of a semi-aromatic polyamide. Considering the filtration efficiency, one can arrange the membranes in the following order: JNF270/JNF90 > JHL > JDL/JTS40 > JTS80 > JNFX.

The properties of the membrane influenced the retention of low molecular weight compounds. For large molecules, for which the main mechanism was spatial blockage, the membrane properties such as surface charge and hydrophilicity were not important. Regarding the SA retention value (as the compound with the lowest MW, pH = 3.5) analyzed during the process, the dense TS80 membrane, made of fully aromatic polyamide, had the best filtration efficiency. Good results were also obtained for the DL membrane. The NFX membrane, which has been described in the literature as a dense aromatic membrane, showed relatively low effectiveness, although the initial retention value was the highest among the membranes tested. This may indicate a significant influence of the adsorption process in the separation of acid low-molecular weight compounds. For loose semi-aromatic membranes such as NF270, the SA separation efficiency was 20%. The efficacy increased significantly with increasing pH.

The dependence of flux and retention values of the tested compounds on the contact angle of the membranes used was analyzed. For some membranes (NFX, TS40 and HL), some correlation was found between filtrate flux and retention and the membrane contact angle. For these membranes, as the contact angle of the membrane increased, the filtrate flux increased, while the initial retention coefficient decreased. Despite a certain scatter of the results data, it was observed that the lower the molar mass of the tested compound, the stronger the influence of the membrane hydrophilicity on the initial retention value. Loose or weakly cross-linked membranes (NF270, NF90) showed high flux values and low retention values, which significantly deviated from the values of the other membranes.

## Figures and Tables

**Table 1:** Structural formulas of selected compounds and their basic properties.

Name	Salicylic Acid (SA)	Acetylsalicylic Acid (ASA)	Amoxicillin (AMX)
Chemical structure	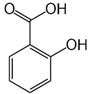	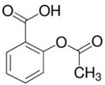	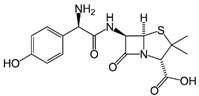
CAS number	69-72-7	50-78-2	26787-78-0
Molar mass (g/mol)	138.12	180.16	365.40 g/mol
Name	Tertacycline (hydrochloride) (TRC)	Diclofenac (DCF) sodium salt	Ibuprofen (IBU) Sodium salt
Chemical structure	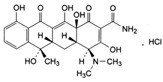	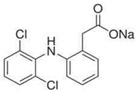	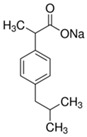
CAS number	64-75-5	15307-79-6	31121-93-4
Molar mass (g/mol)	444.44 g/mol 480.90 g/mol with HCl	318.14 g/mol	228.26 g/mol

**Table 2:** Parameters of membranes used in research.

Characteristic	Type of Membrane						
	NF270	NF90	HL	NFX	DL	TS40	TS80
Polymer	Piperazine polyamide [26,47]	Aromatic polyamide [26,47]	Piperazine polyamide [48,49]	Polyamide *	Modified piperazine polyamide [47]	Piperazine polyamide [26]	Aromatic polyamide [26]
pH range	2–10	2–11	3–9	3–10	2–10	2–11	2–11
MCWO (Da)	200–400	200–400	150–300	~150–300	~150–300	~200	~150
Retention MgSO_4_/NaCl	99.2%/n.d.	99.0%/n.d.	98.0%/n.d.	99%/40%	98%/n.d.	90.0%/40–60%	99.0%/80–90%
Filtrate flux L/m^2·^h/MPa	122–167/0.9	78–102/0.9	66/0.69	32–42/0.76	48/1.52	32/0.76	32.0.76
Producent	Dow Filmtec	Dow Filmtec	GE Osmonics	Synder Filtration ^TM^	GE Osmonics	TriSep^TM^	TriSep^TM^

* Manufacturer data.

**Table 3:** Zeta potential values at pH = 8 and the isoelectric point for selected membranes—literature data.

Type of Membrane	Isoelectric POINT (IEP)	Zeta Potential in pH = 8.0 (mV)	Medium	Source
HL	~3.3	−15	1 mM KCl	[27]
	~3.7	−20	10 mM KCl	[11]
	~4.0	−20	1 mM KCl	[50]
	4.6	−30	1 mM KCl	[26]
	4–4.8	−30	5 mM KCl	[28]
	~4.0	−7	10 mM KCl	[51]
	~4.0	−7	10 mM KCl	[52]
	4.0	−37	1 mM KCl	[53]
	~4.3	~ −27	20 mM NaCl and 1 mM NaHCO_3_	[54]
	~4.0	~ −32	10 mM KCl	[11]
NF90	~3.75	~ −20	10 mM KCl	[36]
NF270	2.5	−52	1 mM KCl	[26]
	2.8–3	−88	5 mM KCl	[28]
	2.8	−25	10 mM KCl	[51]
	3.33	~ −80	1 mM KCl	[35]
	2.9	~ 87	1 mM KCl	[53]
	3.1	~30	10 mM KCl	[11]
TS40	2.5	−52	1 mM KCl	[26]
TS80	2.5	−40	1 mM KCl	[26]
		−14 (pH = 7.0)	10 mM KCl	[42]
DL	<3.3	~−20	1 mM KCl	[27]
	3.69	~−56	1 mM KCl	[35]

**Table 4:** XPS analysis parameters of selected polymer membranes.

Parameters of XPS Analysis	
XPS Analysis for the Full Range of Binding Energy (Widescan) from 1200 eV to 0 eV	High-Resolution XPS Analysis of Elemental Bands (Narrowscan) (Oxygen O 1s, Nitrogen: N 1s, Carbon: C 1s)
Anode power: 30 W	Anode power: 150 W
Spectrum resolution 1 eV	Spectrum resolution 0.1 eV
Scan numbers 2	Scan numbers 3
Pass Energy 160 eV	Pass Energy: 20 eV
Dwell Time 100 ms	Dwell Time 250 ms

**Table 5:** Influence of pH on filtrate flux of tested membranes: pressure 10 bar, temperature 30 °C, SA solution.

Type Membrane		HL	DL	NF90	NF270	TS40	TS80	NFX
Influence pH on flux	pH = 8.0 pH = 3.5	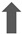	-----	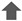	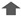	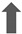	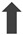	-----
Flux increase (%)		21.4%		6.5%	4%	47%	55%	

**Table 6:** Average values of the contact angle for the three standard liquids and the surface free energy of the selected pure polymer membranes.

Lp.	Type of Membrane	Contact Angle Θ, Deg			Surface Free Energy SFE, mJ/m^2^		
		Θ_W_	Θ_F_	Θ_DIM_	γ^LW^	γ^AB^	γ
1	Virgin Membrane HL	59.0 ± 6.1	51.6 ± 5.2	34.8 ± 3.5	42.1	1.2	43.3
2	Virgin Membrane TS40	28.7 ± 5.9	44.0 ± 4.6	31.5 ± 5.1	43.6	5.1	50.8
3	Virgin Membrane TS80	53.4 ± 7.3	52.5 ± 6.7	32.9 ± 4.1	43.5	7.5	48.3
4	Virgin Membrane NF90	34.0 ± 4.9	38.7 ± 2.8	29.1 ± 3.4	44.6	0.8	45.4
5	Virgin Membrane NFX	17.6 ± 2.8	40.5 ± 4.7	28.3 ± 3.9	44.9	8.1	53.0
6	Virgin Membrane NF270	27.1 ± 0.8	29.6 ± 3.4	24.4 ± 1.1	46.4	3.1	49.5

**Table 7:** The peaks of the FTIR spectra for selected membranes: HL, TS80 and NF90.

Relevant Peaks (cm^−1^)	HL	TS80	NF90	Characteristics
Peaks for polyamide top layer				
1664	-	+	-	Amide I band (C=O stretching—dominant contributor, C–N stretching, and C–C–N deformation vibration in a secondary amide group) [37,67]
1630	+	-	+	Amide I band (poli(piperazineamide)) [37]
1545	-	+	-	Amide II band (N–H in-plane bending and N–C stretching vibration of a –CO–NH– group) [37,67]
Peaks assignable to polysulfone				
1487 1585	+	+	+	Stretching vibrations in the plane of the aromatic ring [37,66]
1350–1280	+	+	+	Asymmetric stretching vibrations of the -SO_2_ groups [37,66]
1245	+	+	+	Asymmetric C-O-C bond vibration of the aryl-O-aryl group [37,66]
1152	+	+	+	Symmetrical vibrations stretching the -SO_2_ groups [37,66]

**Table 8:** Average values of the atomic fraction of individual elements determined on the surface of the tested membranes.

Membrane	Oxygen O (% at.)	Nitrogen N (% at.)	Carbon C (% at.)	O/N
HL	16.00 ± 0.41 13.8 *	11.29 ± 0.26 13.3 *	72.71 ± 0.31 72.9 *	1.42 ± 0.06 1.04
TS80	16.11 ± 0.12	10.66 ± 0.07	73.24 ± 0.09	1.512 ± 0.19
NF90	18.06 ± 0.25 14.0 *	10.90 ± 0.12 12.1 *	71.04 ± 0.22 73.9 *	1.659 ± 0.035 1.16
NF270	16.4 * 16.8 **	12.5 * 10.7 **	71.2 * 72.5 **	1.31 1.57
DL	15.4 *	12.4 *	72.3 *	1.24

* Literature data from [37]. ** Literature data [68].

**Table 9:** Quantitative analysis of the components in the C 1s band for the analyzed membranes.

Membrane	Component Participation (%)		
	C-C/C-H	C-O/C-N	C=O
HL	47.8 ± 0.7	36.6 ± 0.7	15.6 ± 0.13
TS80	56.0 ± 1.0	30.0 ± 0.9	14.0 ± 0.15
NF90	46.8 ± 0.2	36.9 ± 1.6	16.3 ± 0.2

## Data Availability

The data presented in this study are available on request from the corresponding author.

## Supplementary Materials

The following are available online at https://www.mdpi.com/article/10.3390/membranes12020150/s1, Figure S1. Comparative widescan (six analytical fields: p1–p6) for the sample: TS80. Figure S2. Comparative widescan (six analytical fields: p1–p6) for the sample: NF90. Figure S3. Comparative widescan (six analytical fields: p1-p6) for the sample: HL. Figure S4. C 1s region of an XPS spectrum of the membrane TS80. Figure S5. C 1s region of an XPS spectrum of the membrane NF90. Figure S6. C 1s region of an XPS spectrum of the membrane HL.
