# Supplementary material for: Impact of Polymer Membrane Properties on the Removal of Pharmaceuticals
Source: Membranes (Basel). 2022 Jan 26;12(2):150. doi: 10.3390/membranes12020150 (PMC8874440; doi:10.3390/membranes12020150)
Supplement: Supplementary file 1 [file membranes-12-00150-s001.zip › membranes-1482909-supplementary.pdf]

membranes

Article

Impact of the polymer membranes properties on the removal of pharmaceuticals

Renata Żyłła[a*], Magdalena Foszpańczyk[a], Irena Kamińska[a], Marcin Kudzin[a], Jacek Balcerzak[b] and Stanisław Ledakowicz[c]

Citation: Żyła, R.; Foszpańczyk, M.; Kamińska, I.; Kudzin, M.; Balcerzak, J.; Ledakowicz, S. Impact of Polymer Membrane Properties on the Removal of Pharmaceuticals. *Membranes* **2022**, *12*, 150. https://doi.org/10.3390/membranes12020150

Academic Editor: Wolfgang Samhaber and Mohammad Rezaei

Received: 11 November 2021
Accepted: 17 January 2022
Published: 26 January 2022

Publisher's Note: MDPI stays neutral with regard to jurisdictional claims in published maps and institutional affiliations.

Copyright: © 2022 by the authors. Licensee MDPI, Basel, Switzerland. This article is an open access article distributed under the terms and conditions of the Creative Commons Attribution (CC BY) license (https://creativecommons.org/licenses/by/4.0/).

Supplementary material for manuscript:

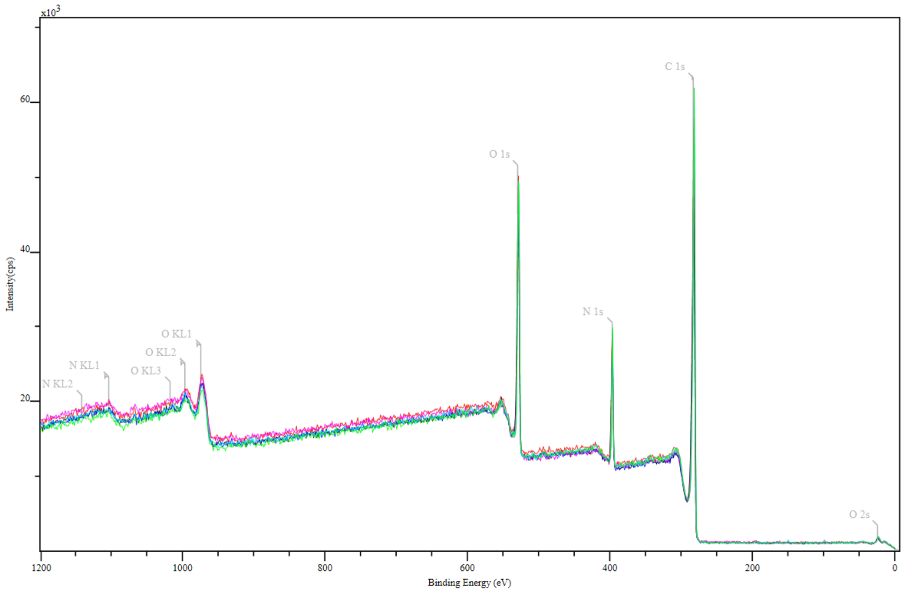

Figure S1. Comparative widescan (six analytical fields: p1-p6) for the sample: **TS80.**

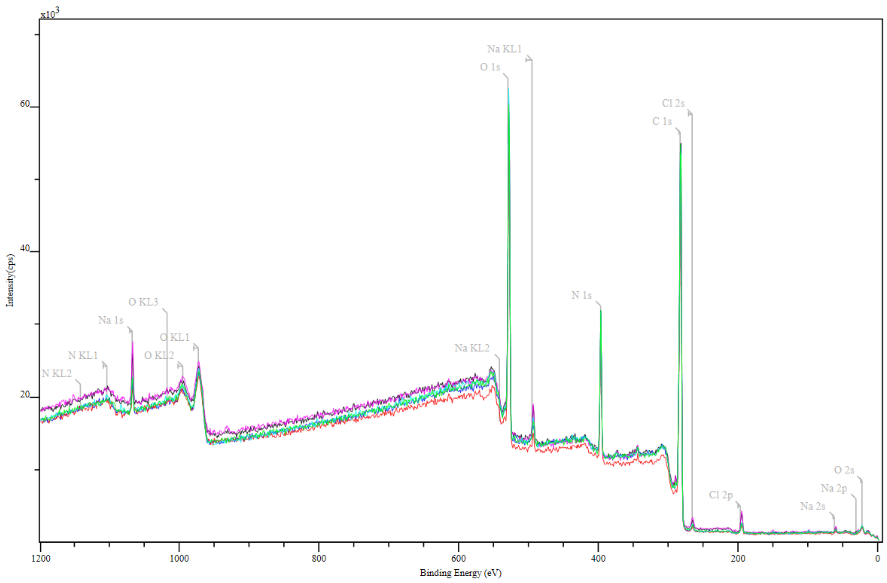

Figure S2. Comparative widescan (six analytical fields: p1-p6) for the sample: **NF90.**

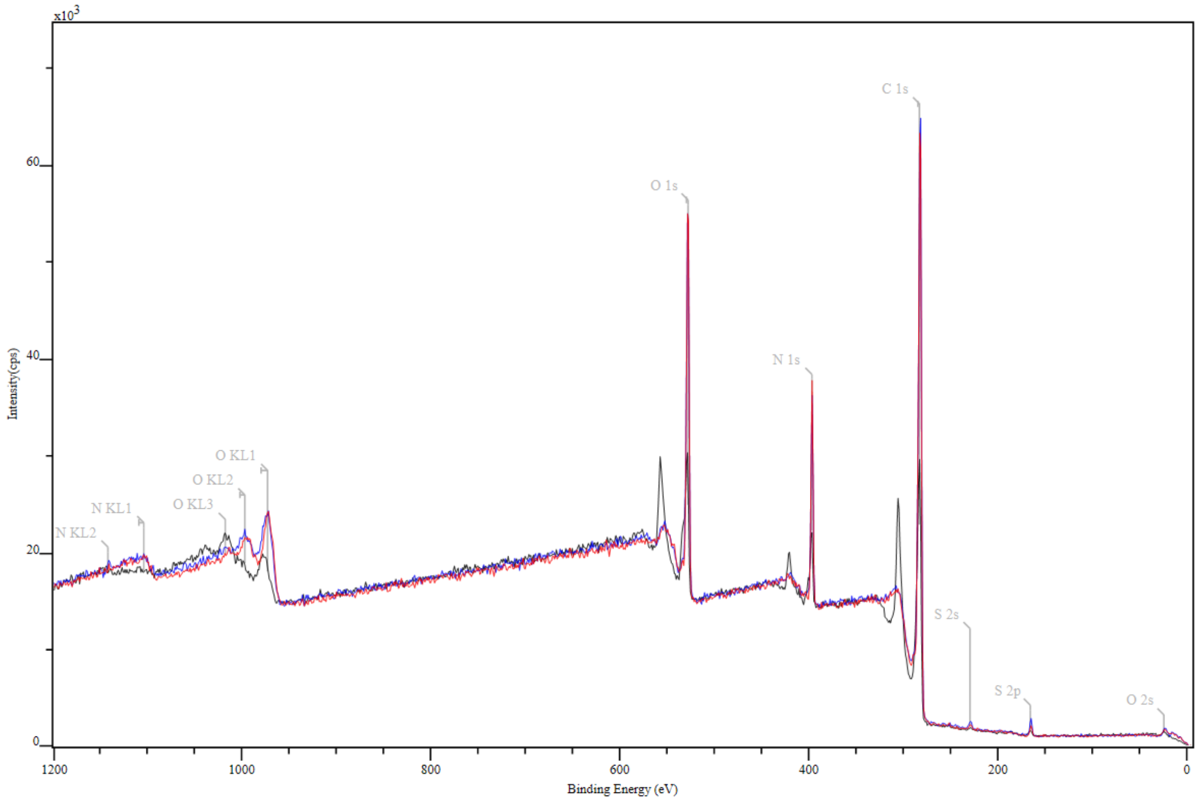

Figure S3. Comparative widescan (six analytical fields: p1-p6) for the sample: **HL.**

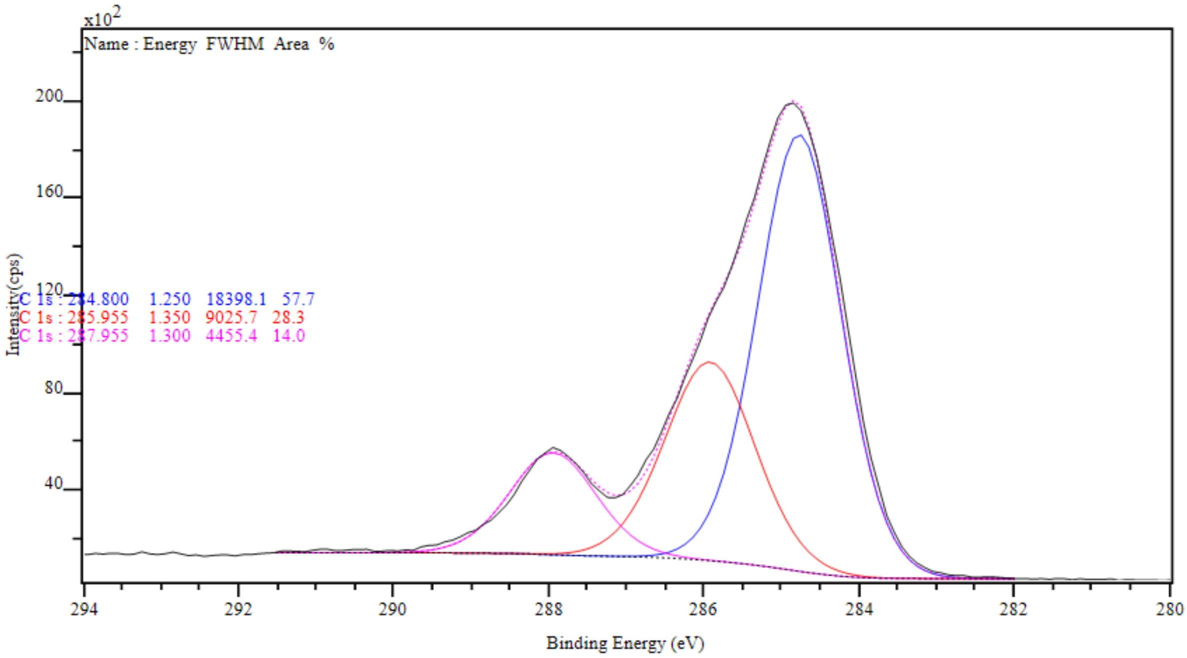

Figure S4. C1s region of an XPS spectrum of the membrane **TS80.**

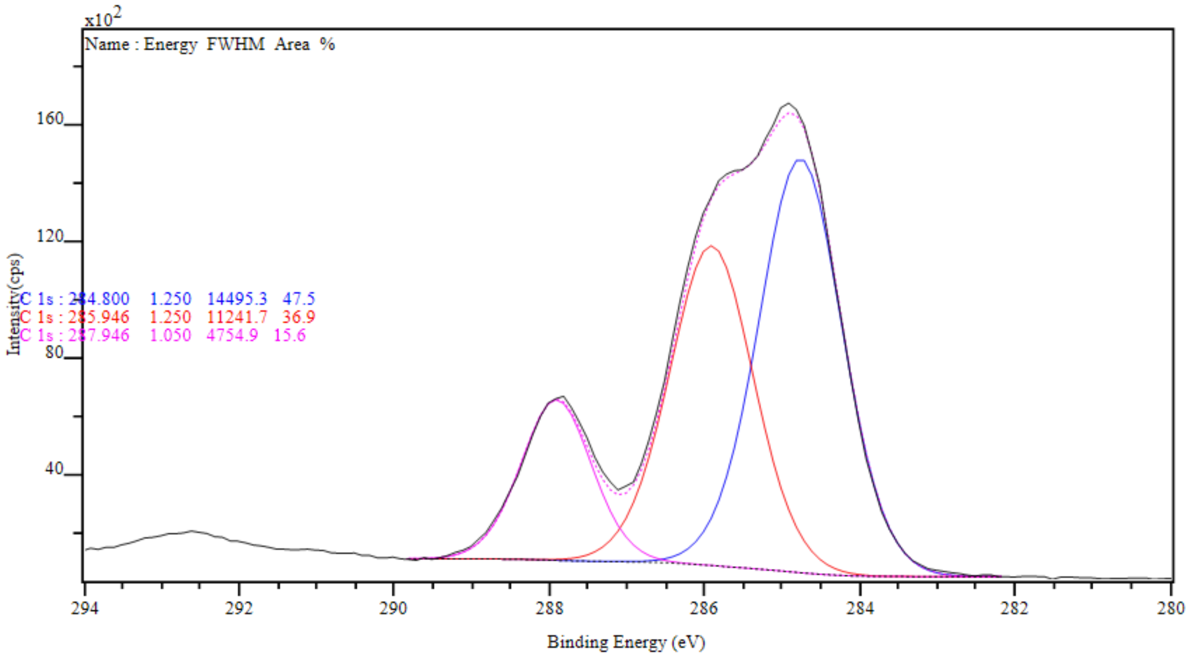

Figure S5. C1s region of an XPS spectrum of the membrane **NF90.**

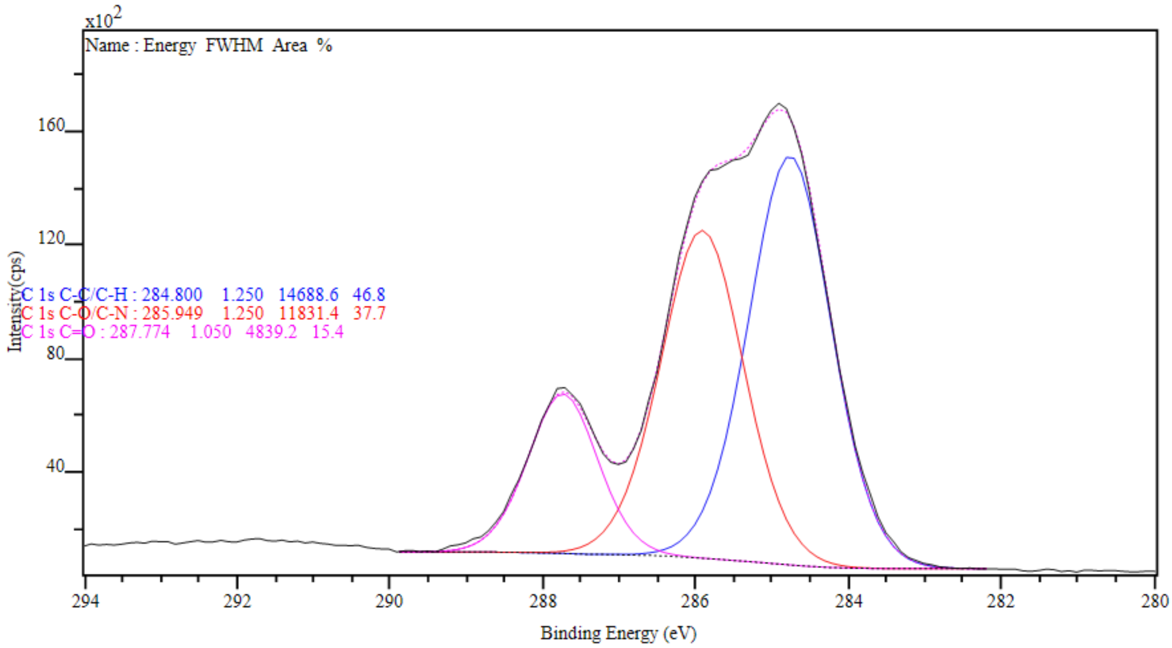

Figure S6. C1s region of an XPS spectrum of the membrane **HL.**
